# Natural language processing analysis applied to COVID-19 open-text opinions using a distilBERT model for sentiment categorization

**DOI:** 10.1007/s00146-022-01594-w

**Published:** 2022-11-21

**Authors:** Mario Jojoa, Parvin Eftekhar, Behdin Nowrouzi-Kia, Begonya Garcia-Zapirain

**Affiliations:** 1 eVIDA Lab, University of Deusto, Bilbo, Spain; 2 Department of Occupational Science & Occupational Therapy, University of Toronto, Toronto, Canada

**Keywords:** Natural language processing, Sentiment analysis, Deep learning, Transformer, DistilBERT

## Abstract

COVID-19 is a disease that affects the quality of life in all aspects. However, the government policy applied in 2020 impacted the lifestyle of the whole world. In this sense, the study of sentiments of people in different countries is a very important task to face future challenges related to lockdown caused by a virus. To contribute to this objective, we have proposed a natural language processing model with the aim to detect positive and negative feelings in open-text answers obtained from a survey in pandemic times. We have proposed a distilBERT transformer model to carry out this task. We have used three approaches to perform a comparison, obtaining for our best model the following average metrics: Accuracy: 0.823, Precision: 0.826, Recall: 0.793 and *F*1 Score: 0.803.

## Introduction

Initially, the novel coronavirus disease 2019 (COVID-19) appeared and reported in December 2019 in China (Chahrour et al. 2020). To reduce the transmission, in 2020, the Chinese government took exceptional steps and lockdown policies. Shortly after that, other countries reported cases of the virus and then it became a global issue (Spina et al. 2020), and the World Health Organization (WHO 2020) announced the pandemic status. At the end of March 2020, in 177 countries, a total of 722,435 positive cases with more than 33,997 mortality were reported (Jhu 2020). Public health authorities, to control the spread of the virus and outbreaks, set different restrictions such as travel bans, social distancing, quarantining, and ban on social gatherings (Bedford et al. 2020). This situation has been linked to mental health stress, morbidity, and mortality (Czeisler et al. 2020). Globally, studies have indicated a significant surge in self-reported anxiety and depression between April and June 2020 in comparison to the same period in 2019. The authors showed that 13% started or heightened substance use to help them to cope with the pandemic, 31% reported anxiety or depression, and 26% showed stress related disorders (CDC 2020; Yan et al. 2020).

This pandemic is an unprecedented incident, which is impacting the health-care, economic, and social systems. This traumatic event has affected society at the individual and population levels. At the individual level, mental health issues have been reported. At the population level, nations experienced economic adversity such as unemployment and poverty, school closures, domestic violence, and inadequate basic food necessities. This situation caused pressure on the global supply chains and health-care systems (Fefferbaum and North 2020).

Fear of contracting the COVID-19, of dying, losing loved ones, uncertainty and fear of future, separation from loved ones, and social discrimination could lead to psychopathological concerns. As a result, this pandemic manifests as a complicated and multifaceted novel event, which is different from natural disasters or previous epidemics such as severe acute respiratory syndrome and Ebola (Brooks et al., 2020).

Educational institutions and training centers also have been affected and shut down, which resulted in social isolation among the younger generation and not being able to socialize with their peers and follow their routine of attending schools. This situation affected their parents’ job as well, since the children stayed home due to closing the childcare centers. The parents were obliged to handle working from home (office work) and providing childcare (double duty). This situation also added to the burnout in parents and caregivers (Zhou et al. 2020).

The use of natural language processing techniques is opening a new horizon of applications. The automatic sentiment analysis capability of the artificial intelligence models is a powerful tool to analyze a large amount of data in short times, with the purpose of obtaining conclusions for decision making. To contribute to be prepared for the next lockdown, we have presented our NLP approach to deal with a large number of open-text answers from questionnaires, instead of using human professionals that are more expensive and slower to perform the sentiment classification.

This paper comprises five sections. Section 1 introduces the theme of the impact of COVID-19 in the daily life of staff and students of universities in different countries. Its aim is also to apply sentiment analysis with artificial intelligence to create strategies to face the lockdown caused by the SARS-CoV-2 virus. The methods used to develop the work are described in the Sect. 2, mainly the distilBERT transformer as a classification model pretrained with GLUE (General Language Understanding Evaluation benchmark) database and fine-tuning with our own data. Section 3 provides the results obtained, which are then discussed in Sect. 4, with the conclusions being drawn in Sect. 5.

## Methods and materials

Invitations were sent to universities across the world to collaborate and support data collection in their local languages during the first and second waves of the COVID-19 pandemic. Inclusion criteria for the study included anyone affiliated with a post-secondary institution (e.g., students, faculty and staff). We also collected data from those not affiliated with a post-secondary institution, but they were not included in this analysis. The questionnaire was available for 6 months (April–November 2020). A similar study was conducted with the Spanish answers in 2021 (Jojoa et al. 2021).

### Data description

The data accumulated for the survey responses are from students, staff and faculty at various universities where each data entry has a survey response of the individual and its respective sentiment. Each dataset of the survey responses contains two major columns, the textual survey response and the sentiment of the respective textual response. The textual response contains the answer of the respondents to the question of how lockdowns during the COVID-19 pandemic affect their mental health.

The data used for the present analysis was collected from 121 countries. The questionnaire was composed of qualitative, quantitative and open-text questions (Nowrouzi-Kia et al. 2022). With the aim of finding key ideas related to the sentiment of the people who answer the questionnaire, we have processed the open-text answers using natural language processing techniques.

A main step in any machine learning task is the labeling process. This activity consists of obtaining a tag from a group of experts who judged, in a subjective way, the sentiment included in each text. Since the paradigm that we have applied to this work is supervised learning, the labeling task is mandatory. To mitigate the risk associated with the bias produced by the experts, we have evaluated the sentiment of each textual answer with a committee of three persons, who evaluated the pertinence of the associated sentiment as two possible categories, positive and negative (Broniatowski 2010).

## Methods

**Fig. 1:**
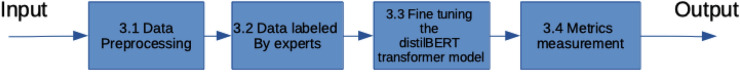
Block diagram of the proposed solution

To carry out the proposed task, the stages presented in the following block diagram were followed in sequential order (see Fig. 1).

### Data pre-processing

Pre-processing the data involves multiple steps that clean and organize the textual data before using the data to fine-tune the machine learning models, and cleaning the data of unnecessary characters in the textual responses, for instance, any special characters while changing formats of the xlsx to CSV, or any redundant formatting in the textual data. Moreover, multiple survey respondents chose not to answer the question about lockdowns impacting their mental health which shows a null value in the textual-response field in our DataFrame. Eliminating such null values ensures no stoppage while fine-tuning the machine learning models. A similar technique of pre-processing the data was replicated for all languages.

To get clean data to be processed using the natural language processing techniques (Kannan Gurusamy 2014), it was mandatory to drop the null fields. Besides, all text was converted to capital letters, to eliminate the case sensitive model issue. On the other hand, text answers with non-clarified ideas were deleted by the experts.

#### Unifying the language of the dataset

The rationale behind the translation of textual responses to English from various languages is to generalize the textual data to a language that we native proficiency speakers could understand and validate the prediction through our understanding of the English language. Fine-tuning individual machine learning models for each language can prove to be a redundant task and would involve different prediction accuracies across the models. Thus, having a unified English language machine learning model maintains consistency throughout the various language data. Most importantly, having a large English dataset with textual data for training that is classified into its respective sentiment incentivized translating various languages to English and generalizing the ML model to learn and predict the sentiment of the English language. Besides, finding sources to train the model specific to languages such as Czech, Russian, Korean, or German could prove to be cumbersome.

For the translation, the use of the Google API was effective, since it allowed the translation of textual responses in multiple batches per API call. An API call is an instruction to the Google Translate application to translate the text embedded in the code of our machine learning model. The Google API allows multiple API calls without any major restrictions on the number of times the API is called, and since it uses Google's Cloud services during the translation there is no need for high-tech computers.

### Data labeled by experts

The transformer-based model (Karpov et al. 2019) is a supervised approach (Conneau et al. 2017) which needs the label of each category per each input text. So, a group of three human experts were met to determine what was the sentiment included in each text. This judgment was performed by human beings, since the ground truth is mandatory for our study. The procedure to carry out this goal was simple, the expert had to read the input text and he/she had to select the perception of sentiment between two categories, positive and negative. Finally, with an agreement of the three judges, the category was labeled to each element of the dataset.

### Fine-tuning distilBERT for sentiment classification

BERT, short for Bidirectional Encoder Representations from Transformers, is a machine learning framework for natural language processing (Lutkevich 2021). “Bidirectional” in BERT represents the model’s ability to read the total sequence of words at once, i.e., each word in an input sentence is processed simultaneously. This mechanism allows the model to learn contextual relations between words (e.g., her in a sentence refers to Jessica), which is useful when predicting the sentiment of an input with multiple sentences. Additionally, BERT’s use in NLP “transfer learning” (Brownlee 2017) enables us to fine-tune the model for sentiment analysis by training with small amounts of data and achieving great results.

However, BERT is highly computational and has a complex structure with millions of parameters (Yates et al. 2021). Thus, fine-tuning the pretrained model requires powerful computational resources, taking us to a more simple, but high-capacity distilBERT model (Sanh 2019). Is a distilled version of the original one BERT, but with less amount of parameters, letting us fine-tune the model in a short time and with medium hardware resources? To carry out this task, the custom dataset consisting of classified English COVID-19 survey responses was used to fine-tune the model for sentiment analysis. Training a machine learning model involves teaching the model to identify positive or negative textual data; thus, the data that we train the model with contains textual data classified according to their sentiment. On the contrary, testing data contains only textual responses since it is the trained model's job to predict each textual response's sentiment in terms of positive and negative. The model accuracy on the test set appeared as 80% which shows that the model can generalize well COVID survey responses in a good manner.

**Fig. 2:**
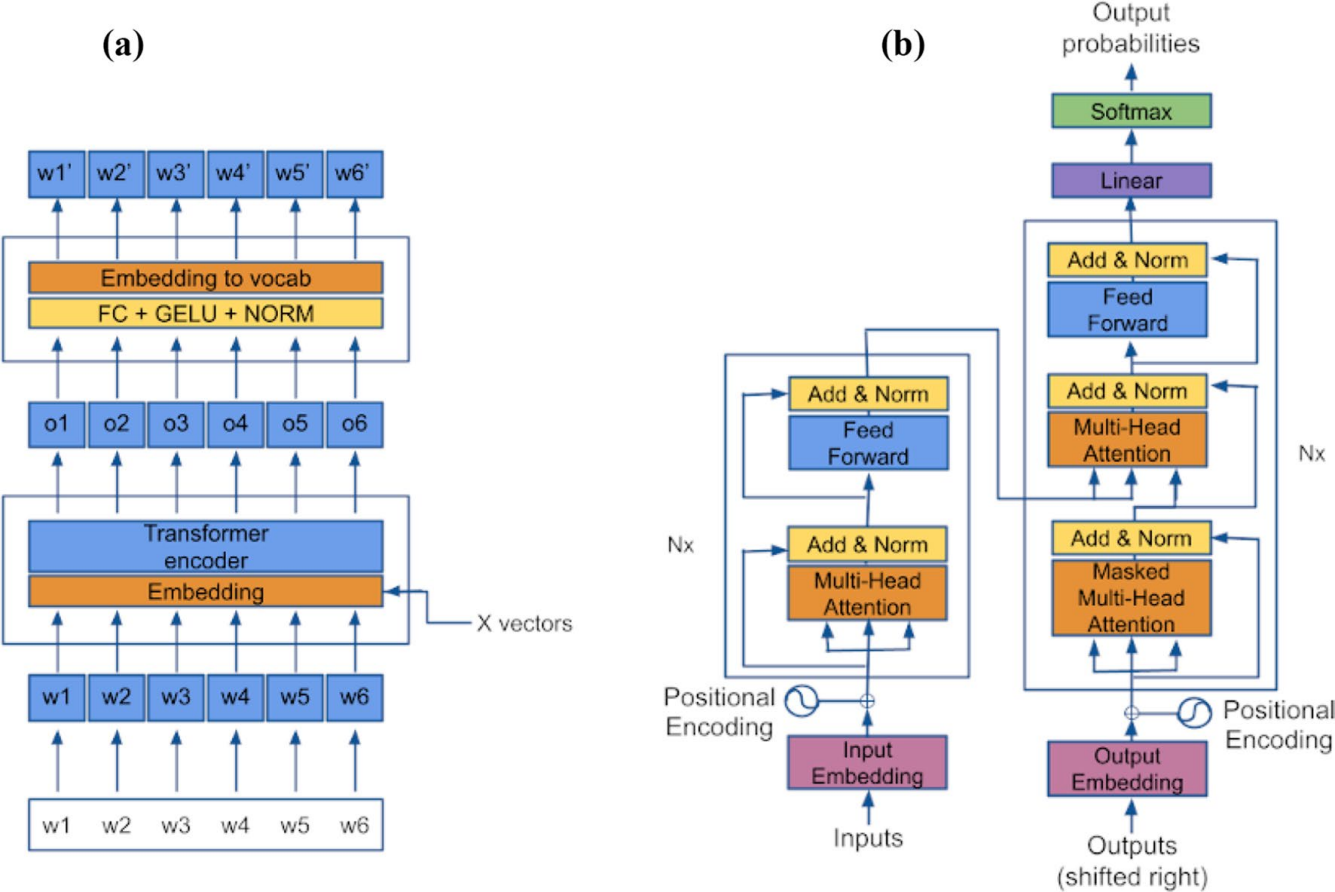
Pretrained transfomer model structure: **a** only encoder, **b** complete model (Tang et al. 2020)

**Fig. 3:**
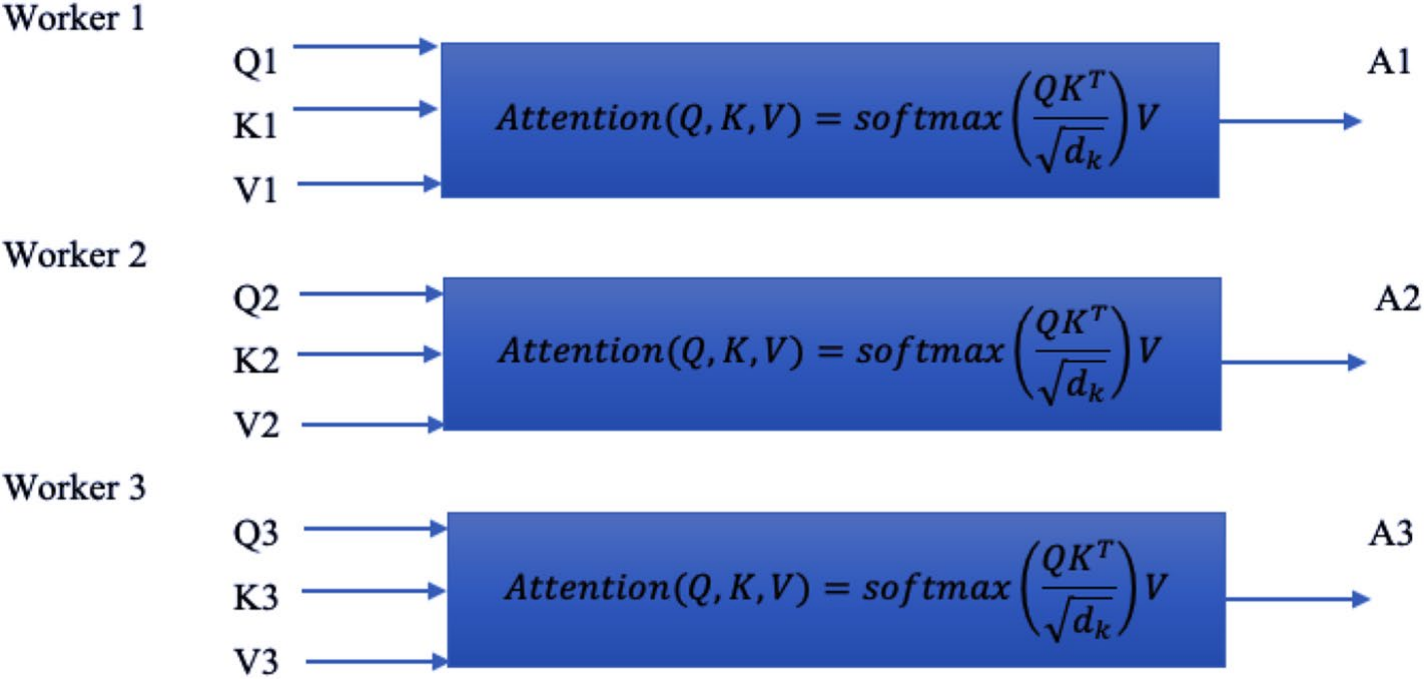
Parallel attention workers for multi-attention blocks

The transformer model is shown in Fig. 2b, where the encoder and decoder structures are included to perform a classification task. However, the power of this kind of structures is based on the principle of attention (Vaswani et al. 2017), this is:

$${\text{Attention }}\left( {Q,K,V} \right) = {\text{softmax}}\left( {\frac{{QK^{{\text{T}}} }}{{\sqrt {d_{k} } }}} \right)V {\text{Ec}}. 1,$$

where *Q* is the query vector, *K* is the key vector, and *V* the value vector.

$$\begin{aligned} Q & = X*W_{q} , \\ K & = X*W_{k} , \\ V & = X*W_{v} , \\ \end{aligned}$$

where *X* are the vectors obtained at the output of the embedding and *W* the weights associated with each layer of the feed-forward neural network of each decoder. In the same way, the multi-head attention could be modeled as different single attention blocks in parallel with different workers. Figure 3 shows this idea.

The advantage of using this approach is in finding correlation between each word. The intuition behind multi-head attention is that it allows us to attend to different parts of the sequence differently each time. This means that the model can better capture positional information, because each head will attend to different segments of the input. The combination of them will give a more robust representation. On the other hand, each head will capture different contextual information as well, by correlating words in a unique manner (Ho et al. 2019).

Based on this idea, the fine-tuning (Sanh et al. 2019) process of the model is to adjust and update the weights of the feed-forward neural networks related to the attention layers of the distilBERT transformer with the information of the new context to be added, in terms of geting the new specific capacity, desired for our study.

### Metrics measurement

The classification task must be measured in the same way as the classifiers. In our case, the model is based on a binary classification. To measure the performance, we have decided to use the typical metrics such as Accuracy, Precision, Recall and *F*1 score (Wang, 2018).

$$\begin{gathered} {\text{Accuracy }} = \frac{{{\text{TP}} + {\text{TN}}}}{{{\text{TP}} + {\text{TN}} + {\text{FP}} + {\text{FN}}}}\quad {\text{Ec}}.{\text{1 Formula of Accuracy,}} \hfill \\ {\text{Precision}}\left( i \right) = \frac{{{\text{TP}}\left( i \right)}}{{{\text{TP}}\left( i \right) + {\text{FP}}\left( i \right)}}\quad {\text{Ec}}.{\text{2 Formula of Precision,}} \hfill \\ {\text{Recall}}\left( i \right) = \frac{{{\text{TP}}\left( i \right)}}{{{\text{TP}}\left( i \right) + {\text{FN}}\left( i \right)}}\quad {\text{Ec}}.{\text{3 Formula of Recall,}} \hfill \\ F{\text{1 score}}\left( {\text{i}} \right) \, = 2 \times \frac{{{\text{Precision}}\left( i \right) \times {\text{Recall}}\left( i \right)}}{{Precision\left( i \right) + {\text{Recall}}\left( i \right)}}\quad {\text{Ec}}.{\text{4 Formula of F1 score}}{.} \hfill \\ \end{gathered}$$

These metrics are the most used to measure the performance of a classifier. Accuracy gives us an idea of the general behavior of the model, but does not take into account the bias produced by the unbalanced dataset. So, we complemented the measurement process including Precision, which is the positives rate related to false positives, recall the positives rate related to false negatives and F1 score which is the second harmonic mean mixing the precision and recall metrics.

## Results and discussion

This section presents the results obtained after applying the distilBERT model to the data. This model was fine-tuned with the database described in the section “Materials”. With the fine-tuned model, a classification was performed in two categories of sentiment present in each of the analyzed text. It is very important to highlight that the output of the transformer is a label: negative represents the worst sentiment in the text and positive the best one.

The classifier pretrained with GLUE (General Language Understanding Evaluation benchmark) (Liu et al. 2020) dataset was fine-tuned in three different approaches. The hyperparameters used for the first approach are showed in Table 1.

**Table 1:** Hyperparameters used for the first approach

Hyperparameters	Value
Total number of training epochs	num_train_epochs = 6
Batch size per device during training	per_device_train_batch_size = 16
Batch size for evaluation	per_device_eval_batch_size = 64
Number of warmup steps for learning rate scheduler	warmup_steps = 500
Strength of weight decay	weight_decay = 0.01

The first approach used splitted dataset in 80% of training and 20% of testing, without taking into account the balance between positive and negative categories. The confusion matrix obtained is presented in Table 2.

**Table 2:** Confusion matrix obtained for the first approach in the sentiment detection task

	Predicted			Amount of Positives/Negatives
	Class	Positives	Negatives	Total
Real	Positives	69	56	125
	Negatives	17	187	204

The hyperparameters obtained using the grid search method allowed us to get the best performance for this distribution of data.

Table 2 shows that sentences with negative sentiment have a high tendency to be well classified. However, positive sentences have a lot of incorrect classifications. This will allow us to conclude the necessity of applying a second approach with a subset more balanced data.

The second approach used a subset composed by in a perfect balance of 350 positive cases and 350 negatives cases. The confusion matrix obtained is presented in Table 3. The hyperparameters used are shown in Table 4.

**Table 3:** Confusion matrix obtained for the second approach in the sentiment detection task

	Predicted			Amount of positives/negatives
	Class	Positives	Negatives	Total
Real	Positives	85	40	125
	Negatives	19	185	204

**Table 4:** Hyperparameters used for the second approach

Hyperparameters of model 2	Value
Total number of training epochs	num_train_epochs = 8
Batch size per device during training	per_device_train_batch_size = 16
Batch size for evaluation	per_device_eval_batch_size = 64
Number of warmup steps for learning rate scheduler	warmup_steps = 500
Strength of weight decay	weight_decay = 0.01

The hyperparameters obtained for this approach show us a difference only in the number of epochs.

Table 5 shows that sentences with negative sentiment continue with a high tendency to be well classified. However, positive sentences now are better classified than that with the last approach.

**Table 5:** Hyperparameters used for the second approach

Hyperparameters of model 2	Value
Total number of training epochs	num_train_epochs = 4
Batch size per device during training	per_device_train_batch_size = 16
Batch size for evaluation	per_device_eval_batch_size = 64
Number of warmup steps for learning rate	warmup_steps = 500
Strength of weight decay	weight_decay = 0.01

The third approach used splitted dataset in 90% of training and 10% of testing percentage in the not balanced dataset. The hyperparameters used are shown in Table 5. The confusion matrix obtained is presented in Table 6.

**Table 6:** Confusion matrix obtained for the third approach in the sentiment detection task

	Predicted			Amount of Positives/negatives
	Class	Positives	Negatives	Total
Real	Positives	40	20	60
	Negatives	8	90	98

Similar to the other cases, the only hyperparameter that changed was the number of epochs to fine-tune the model.

Table 6 shows a better performance, but the amount of data to test the model was reduced by 50% without a high difference in performance compared with the other two models.

Based on the Eqs. 1, 2, 3 and 4, we calculated the metrics for the “negative sentiment” and “positive sentiment” classes. Finally, we have averaged both to get an objective perspective of the performance of our sentiment detector/classifier based on the distilBERT transformer.

The accuracy value for classifying sentences with negative sentiment is higher for the third approach, although with a value very similar to that of the second approach. Furthermore, the first model has a very good recall for the negative class (Table 7), but a very poor recall for the positive class (Table 8). Therefore, we conclude that the first model is not valid, and either of the other two is. Bold values are the best obtained for each model.

**Table 7:** Results obtained based on “negative sentiment” class as the reference class

Approach	Accuracy	Precision	Recall	*F*1 score
1st (data 80–20)	0.778	0.770	0.917	0.837
2nd (data 50–50)	0.821	**0.822**	0.907	0.862
3th (data 90–10)	**0.823**	0.818	**0.918**	**0.865**

**Table 8:** Results obtained based on “positive sentiment” class as the reference class

Approach	Accuracy	Precision	Recall	F1 score
1st (data 80–20)	0.778	0.802	0.552	0.654
2nd (data 50–50)	0.821	0.817	0.680	**0.742**
3th (data 90–10)	**0.823**	**0.833**	**0.667**	0.741

Now, we have decided to calculate the average for each of the metrics. This is done to observe the behavior of the classifier for an unbalanced dataset. From this, we have concluded that model 3 performs the best. However, as is evident, the values of the metrics are very close to model 2 (Table 9).

**Table 9:** Average of the results obtained for “negative sentiment” and “positive sentiment” classes

Approach	Accuracy	Precision	Recall	F1 Score
1st (data 80–20)	0.778	0.786	0.734	0.745
2nd (data 50–50)	0.821	0.820	0.793	0.802
3th (data 90–10)	**0.823**	**0.826**	**0.793**	**0.803**

Therefore, including an additional criterion, which corresponds to the amount of data used to test the classifier, we conclude that approach 2 is the best for the task of detecting positive or negative sentiment in the input data.

As a discussion, distilBERT is a simpler model of BERT transformer with the same capacity of the bigger one. However, we could be concerned about the idea that reducing the number of parameters could affect the general performance of the model. In a paper by Sanh et al. (2019), Liu et al. (2020), and Yu et al. (2021), we find that distilBERT has presented a very good behavior regarding lower computational complexity. For our application, this is an advantage, because the amount of available data is limited, so this condition leads us to prefer models with low number of parameters but with high capacity.

On the other hand, the use of Google Api to perform the automatic translation of the words could be a risk at this point, since the final result of our system is highly sensitive to the words used to express the ideas in the input text. In Qiu et al. (2020), the authors show how the automatic language translation is performed for a natural language processing model and how the use of encoder–decoder approach generates an objective sequence in the new desired code, in this case, another language. Our model has learned from a general purpose dataset and it was tuned for the sentiment analysis detection, using the text answers of our surveys. However in a previous stage, an automatic translation process of some inputs was carried out using a Google service. It is clear that Google uses NLP models to perform this task, so a little black box appears in this point and should be addressed to have a whole understanding of each stage of our research work.

The COVID-19 pandemic and the subsequent lockdown measures enacted by various states impacted the mental health and well-being of students, faculty and staff. Specifically, we found that the lockdown impacted the social and quality of life of students, and faculty across the world. We postulate that additional mental health supports need to be provided to support those struggling as a result of the pandemic (Aqeel et al. 2021; Cao et al. 2020; Abbas et al. 2019).

Our proposed approach is a milestone for automatic analysis of open-text answers from a COVID-19 lockdown questionnaire. Since the decision making needs a faster analysis, the use of a large number of human experts is expensive for government and mental health institutions. Our model proposes a solution to this issue, since the artificial intelligence models can learn from experts and apply the learned knowledge to resolve problems such as the ones faced in our work, but in shorter times. The beneficiaries are all stakeholders involved in the lockdown situation.

## Conclusion

The seq2seq model allows us to find sentiment information inside the analyzed texts. However, a high amount of data is needed to carry out this task, since the transformer structure has millions of parameters inside. So, fine-tuning is a good alternative to reduce the computational cost of the train from scratch for the sentiment analysis model.

Sentiment analysis may be one approach to provide support for students and faculty as we prepare for future public health crises. One of the challenges of this study was that we rapidly mobilized the launch of the survey and as a result the psychometric properties of the survey instrument were not rigorously evaluated.

The main limitation of the present work is the use of a pretrained model as a base to fine-tune a specific model, since this situation could cause bias regarding the data used to train the model from scratch. Nadeem et al. (2004) Lovering et al. (2020), Sahlgren and Olsson (2019) describe how in language models, the bias appears after a fine-tuning process applied to perform a specific task. However, they propose the fine-tuning technique as an option where the amount of data is limited and the computational cost is an issue to be dealt with in the research process. Besides, the double sense, sarcasm, metaphors are a key issue to be addressed in future works, since this is a problem of the state-of-the-art natural language models.

Finally, the model is limited in relation to the amount of data, since we could not train our own embedding to codify the input words as vectors. It is necessary for more sensitive works to build an embedding to get more accurate representations, regarding the context, of the inputs used in our study.

Future research should consider the long term. The long-term implications of the lockdown are unknown and need to be examined using cohort and longitudinal studies. Combined with machine learning techniques, the findings may provide improved mental health interventions that universities and other educational institutions may use to provide supports for students and faculty who are struggling with their mental health (Nowrouzi-Kia, 2022).

It is very important to consider that working with transfer learning has advantages and disadvantages. So, to reduce the bias caused by the limited amount of data to fine-tune the models, the data collection is mandatory for future research works. Besides, once the data is collected, we can train a specific embedding and a distilBERT model from scratch to perform the sentiment analysis task.

## Data Availability

Data were obtained from (Osipenko, 2021) Osipenko The LockedDown Survey. Available online: https://www.healthbit.com/the-lockeddown/ (accessed on 28 May 2021).
